# Supplementary material for: Coalescence of carbon nanotubes while preserving the chiral angles
Source: Nat Commun. 2025 Feb 5;16:1093. doi: 10.1038/s41467-025-56389-6 (PMC11799306; doi:10.1038/s41467-025-56389-6)
Supplement: Supplementary file 1 — Supplementary Information [file 41467_2025_56389_MOESM1_ESM.pdf]

Supplementary Information

Coalescence of carbon nanotubes while preserving the chiral angles

Akira Takakura[1], Taishi Nishihara[1], Koji Harano[2,3], Ovidiu Cretu[2], Takeshi Tanaka[4], Hiromichi Kataura[4], Yuhei Miyauchi[1]*

[1]*Institute of Advanced Energy, Kyoto University, Uji, Kyoto 611-0011, Japan.*

[2]*Center for Basic Research on Materials, National Institute for Materials Science, Tsukuba, Ibaraki 305-0044, Japan*

[3]*Research Center for Autonomous Systems Materialogy (ASMat), Institute of Integrated Research, Institute of Science Tokyo, Yokohama, Kanagawa 226–8501, Japan*

[4]*Nanomaterials Research Institute, AIST, Tsukuba, Ibaraki 305-8565, Japan.*

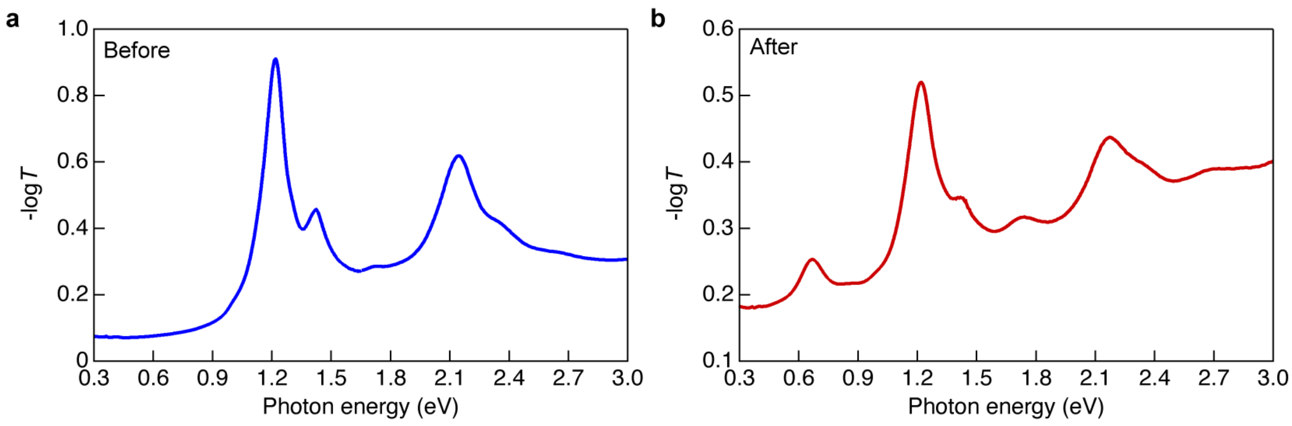

Supplementary Fig. 1 | Broadband optical absorption spectra. (**a,b**) Optical absorption (−log *T*, where *T* is transmittance) spectra of (6,5) nanotube membranes before (**a**) and after (**b**) the vacuum heat treatment. Source data are provided as a Source Data file.

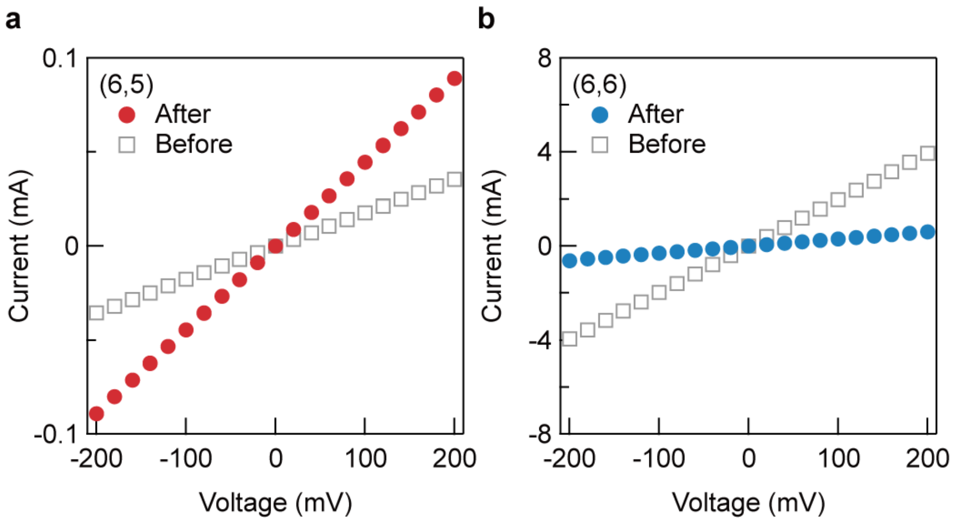

Supplementary Fig. 2 | Changes in electric resistance. (a,b) Current–voltage plot for semiconducting (6,5) (**a**) and metallic (6,6) (**b**) carbon nanotube membranes. The open rectangles and filled circles represent the membranes before and after heat treatment at 900 ºC, respectively. Source data are provided as a Source Data file.

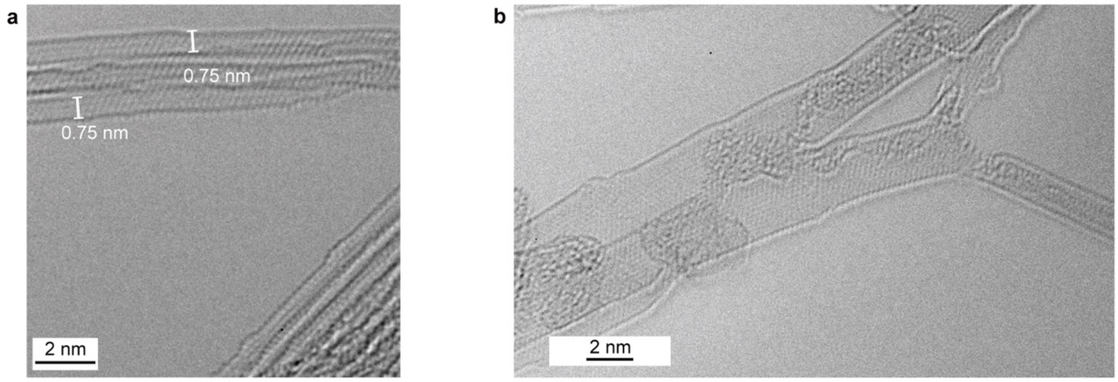

Supplementary Fig. 3 | Transmission electron microscope (TEM) image of the carbon nanotube membrane before and after heat treatment. (**a**)TEM image of the (6,5) nanotubes with the diameter of 0.75 nm before the heat treatment. (**b**) Junction of three nanotubes with different diameters found after the heat treatment: (6,5) and (12,10) nanotubes, and one with a diameter close to that of (18,15) (almost three times the diameter of the (6,5) nanotube). These TEM images were obtained using aberration-corrected TEM (JEOL, JEM-ARM200F) with the acceleration voltage of 60 kV.

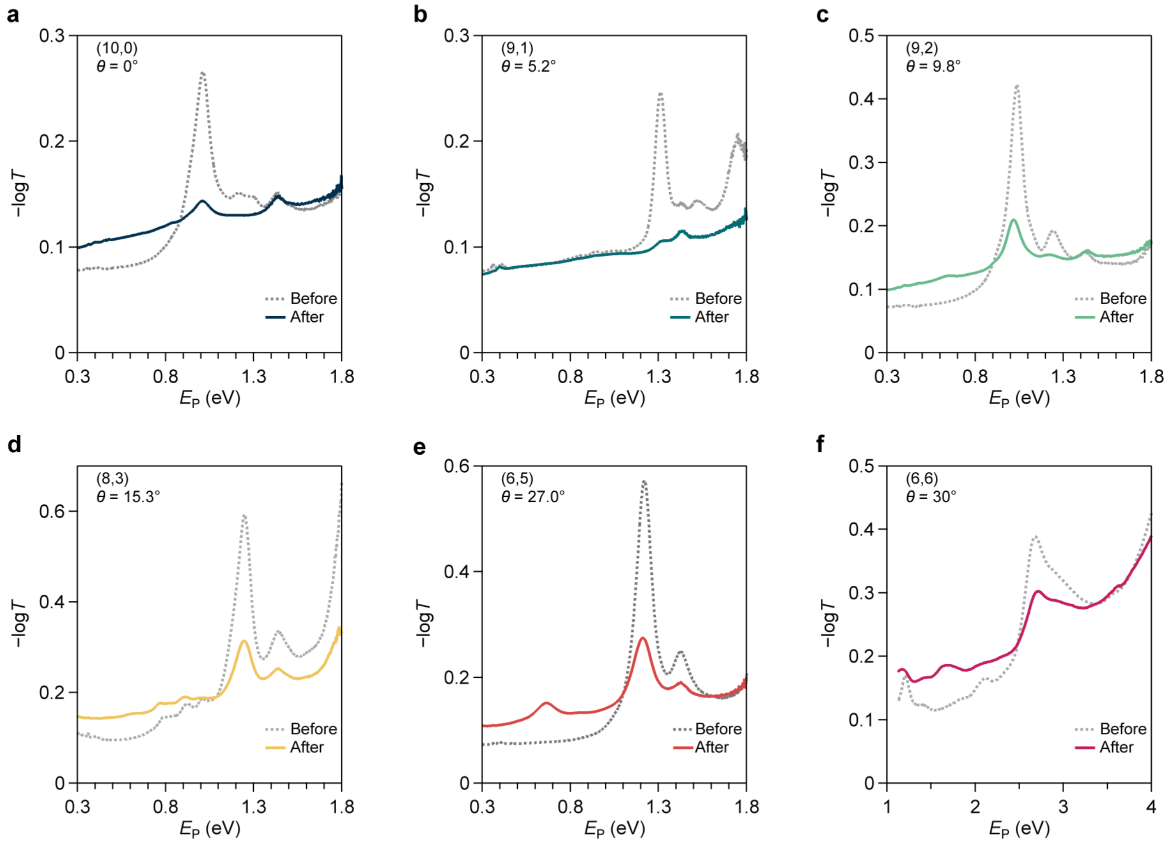

Supplementary Fig. 4 | Optical absorption spectra before and after the vacuum heat treatment at 1000 ºC. (a–f) Optical absorption (−log T, where T is transmittance) spectra of the carbon nanotube membranes composed of (10,0) (**a**), (9,1) (**b**), (9,2) (**c**), (8,3) (**d**), (6,5) (**e**), and (6,6) (**f**) nanotubes before and after the vacuum heat treatment at 1000 ºC. E_P, photon energy. Source data are provided as a Source Data file.

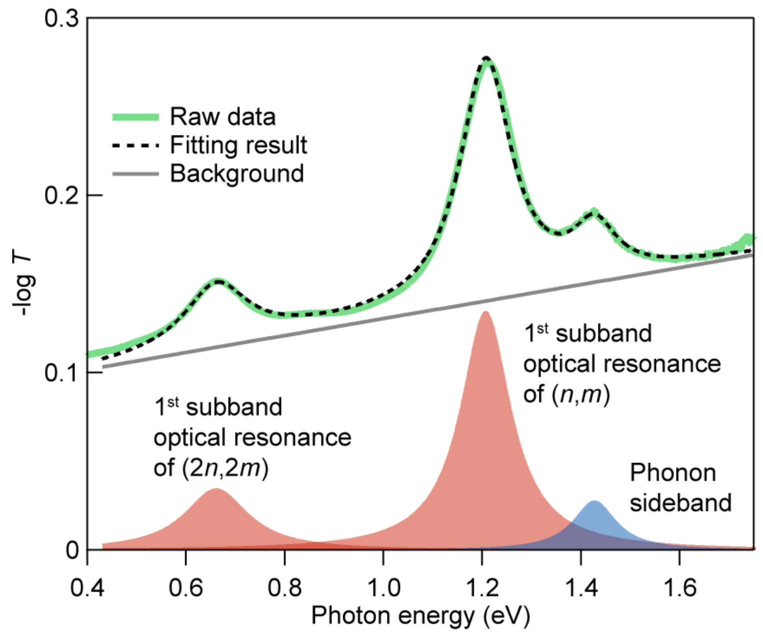

Supplementary Fig. 5 | Fitting procedures. Experimental data (light green curve) and the fitting curve (black broken line). The fitting components are the grey line (baseline) and the red and blue filled areas. Source data are provided as a Source Data file.

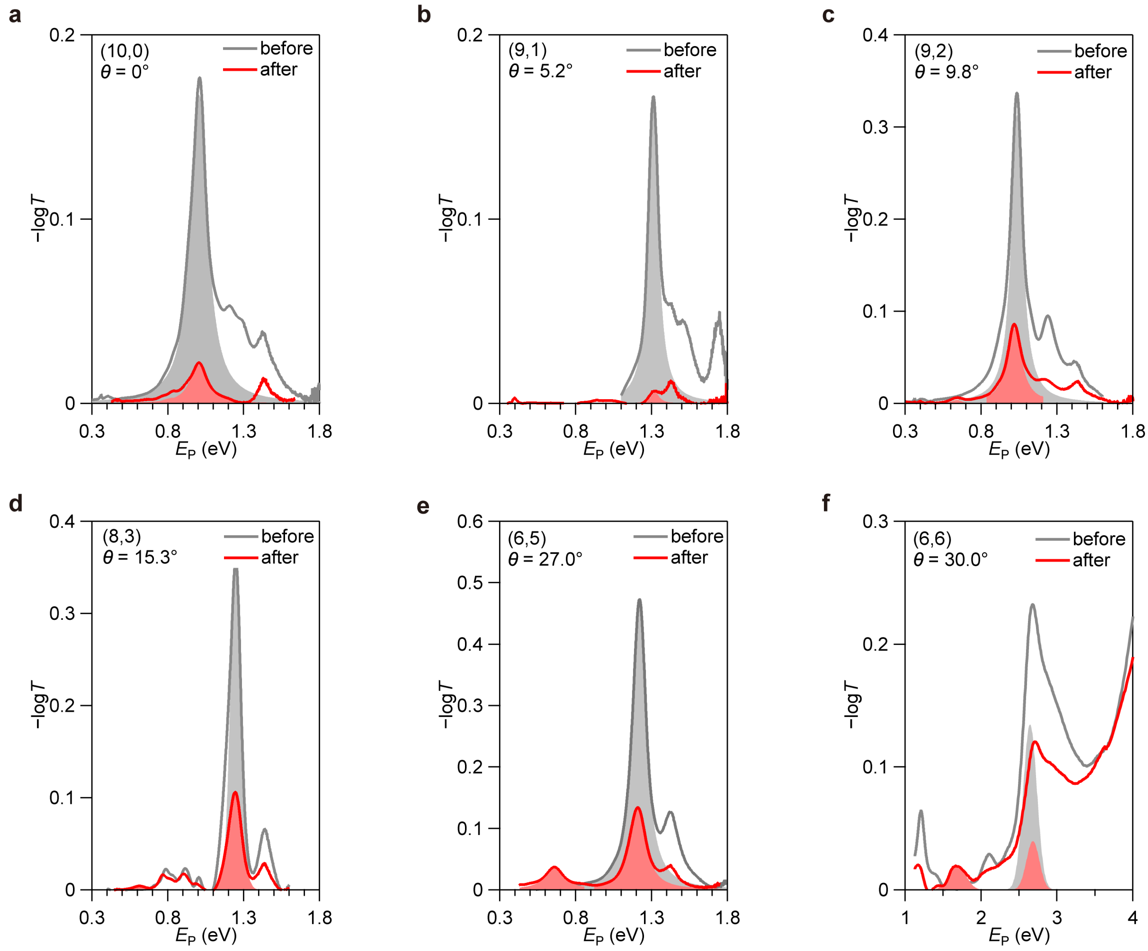

Supplementary Fig. 6 | Absorption spectra with the exclusion of the background baseline. (a–f) Spectra of the carbon nanotube membranes composed of (10,0) (**a**), (9,1) (**b**), (9,2) (**c**), (8,3) (**d**), (6,5) (**e**), and (6,6) (**f**) nanotubes before and after the vacuum heat treatment at 1000 ºC. Coalescence efficiencies were determined by evaluating the area of the gray and red shaded regions. E_P, photon energy. Source data are provided as a Source Data file.

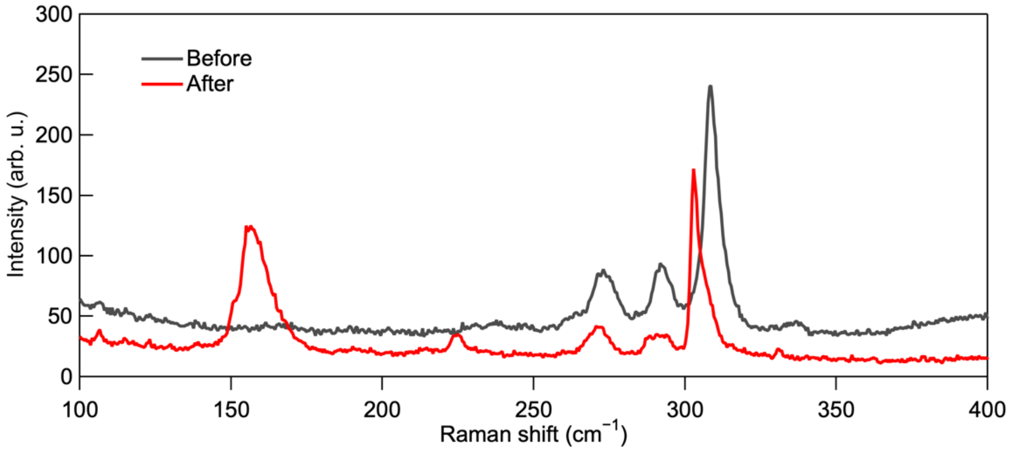

Supplementary Fig. 7 | Radial breathing mode feature. The Raman spectra of the (6,5) nanotube membrane before and after heat treatment. Source data are provided as a Source Data file.

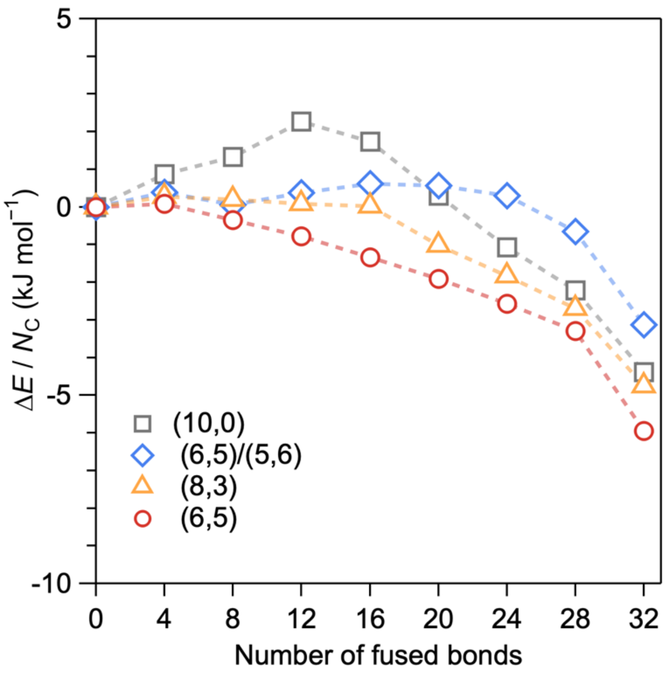

Supplementary Fig. 8 | Energy change of partially coalesced nanotubes. Energy difference ΔE of partially coalesced nanotubes relative to that of the initial two nanotubes normalized by the number of carbon atoms N_C in the calculation, plotted as a function of the number of cleaved and newly formed bonds along the zigzag direction, as calculated using molecular mechanics simulations. Source data are provided as a Source Data file.

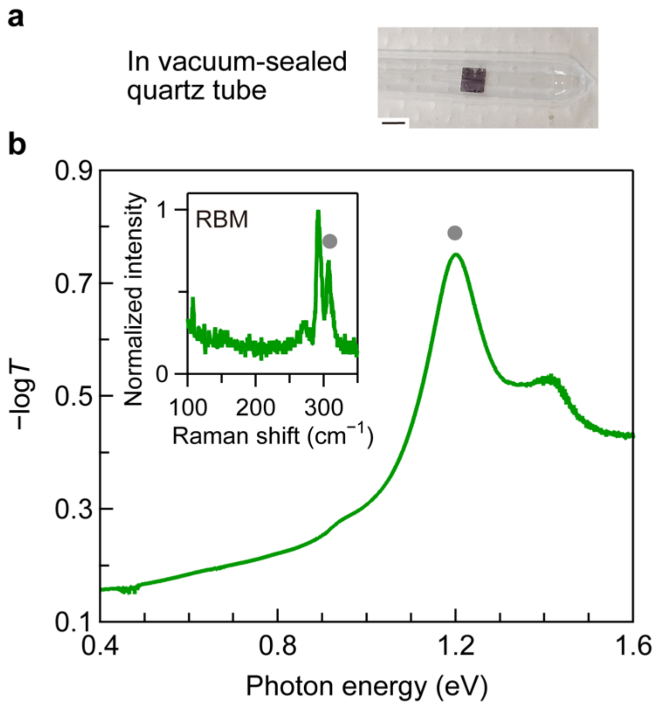

Supplementary Fig. 9 | Heat treatment in a high-vacuum-sealed quartz tube. (**a**) Photograph of the (6,5) nanotube membrane on a sapphire substrate placed in a high-vacuum-sealed quartz tube. Scale bar, 5 mm. (**b**) Optical absorption of the (6,5) nanotube membrane on a sapphire substrate placed in a high-vacuum-sealed quartz tube after the reaction. The inset shows the Raman spectrum in the radial-breathing-mode (RBM). The gray filled circles indicate the peaks for (6,5) species. Source data are provided as a Source Data file.

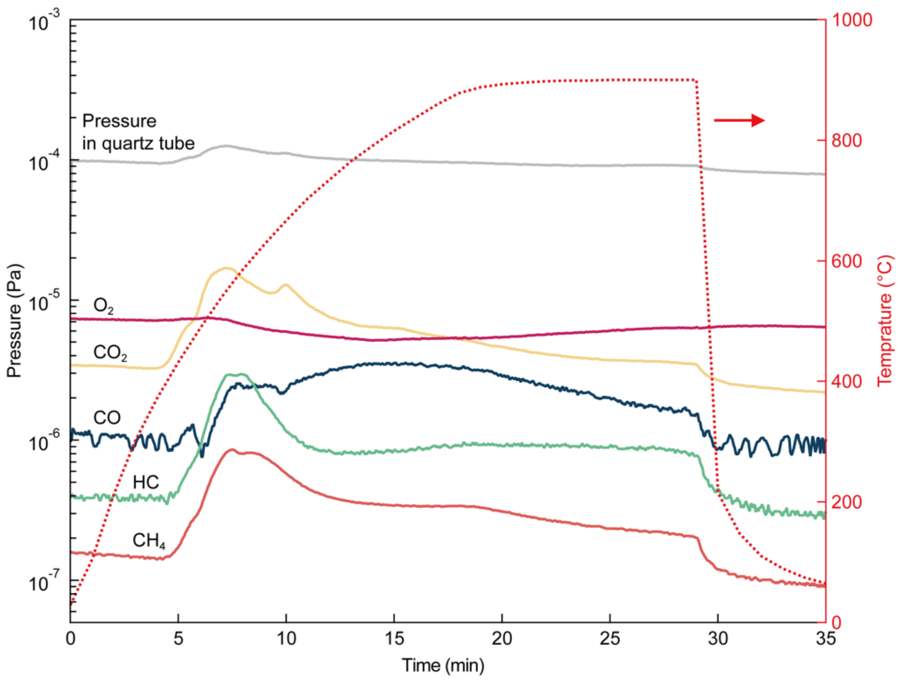

Supplementary Fig. 10 | Gas composition. Partial pressure (left axis) in the quartz tube and temperature of the quartz tube (right axis). HC, hydrocarbon. Source data are provided as a Source Data file.

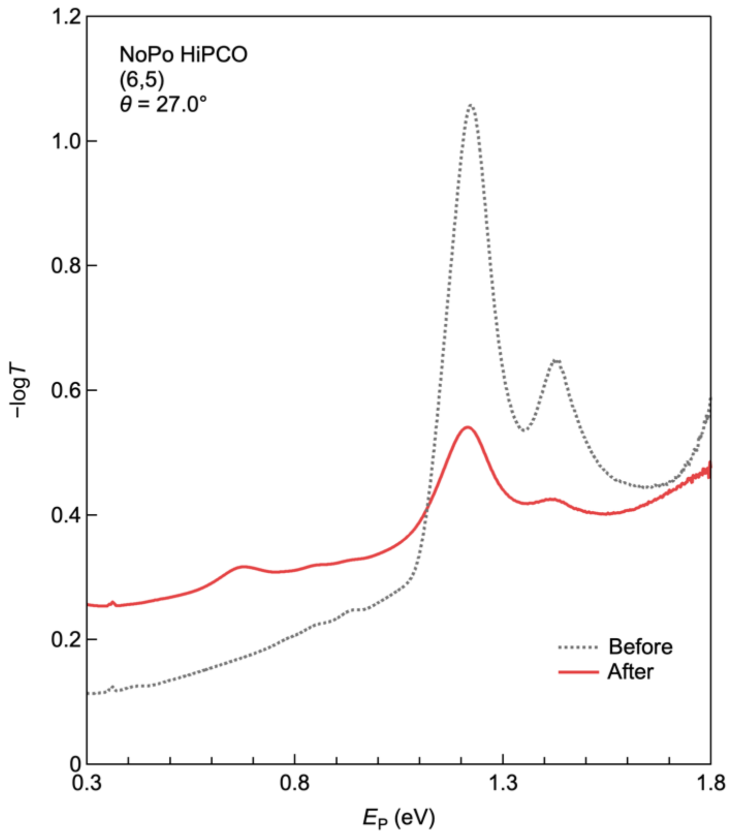

Supplementary Fig. 11 | Coalescence of the (6,5) nanotubes separated from NoPo HiPCO sample. Optical absorption spectra of the (6,5) nanotube membrane from NoPo HiPCO before and after the vacuum heat treatment at 1000 ºC. Source data are provided as a Source Data file.
